# Evolutionary Trails of Plant Group II Pyridoxal Phosphate-Dependent Decarboxylase Genes

**DOI:** 10.3389/fpls.2016.01268

**Published:** 2016-08-23

**Authors:** Rahul Kumar

**Affiliations:** Repository of Tomato Genomics Resources, Department of Plant Sciences, University of HyderabadHyderabad, India

**Keywords:** PLP decarboxylase, evolution, plants, phylogeny, fruits, gene expression, potato, gene complement

## Abstract

Type II pyridoxal phosphate-dependent decarboxylase (PLP_deC) enzymes play important metabolic roles during nitrogen metabolism. Recent evolutionary profiling of these genes revealed a sharp expansion of histidine decarboxylase genes in the members of Solanaceae family. In spite of the high sequence homology shared by PLP_deC orthologs, these enzymes display remarkable differences in their substrate specificities. Currently, limited information is available on the gene repertoires and substrate specificities of PLP_deCs which renders their precise annotation challenging and offers technical challenges in the immediate identification and biochemical characterization of their full gene complements in plants. Herein, we explored their evolutionary trails in a comprehensive manner by taking advantage of high-throughput data accessibility and computational approaches. We discussed the premise that has enabled an improved reconstruction of their evolutionary lineage and evaluated the factors offering constraints in their rapid functional characterization, till date. We envisage that the synthesized information herein would act as a catalyst for the rapid exploration of their biochemical specificity and physiological roles in more plant species.

## Introduction

Pyridoxal 5′-phosphate (PLP), one of the active prosthetic group of vitamin B_6;_ is a coenzyme with unequaled catalytic versatility. This is involved in a plethora of biochemical reactions like transamination (transfer of amino groups), decarboxylation (removal of a carboxyl group at the β- or γ-carbon), deamination (removal of an amine group), interconversion of L and D-amino acids, and racemization. PLP-dependent enzymes are mainly involved in the regulation of biosynthesis of amino acids, amino acid-derived metabolites, amino sugars and other amine-containing compounds (Facchini et al., 2000; Eliot and Kirsch, 2004). It has been found that the enzymatic versatility of these enzymes is achieved by the covalent binding of PLP group to an amino group of an active lysine in their substrates (John, 1995). Depending on their protein structures, all PLP-dependent enzymes have been classified in at least five structural groups (Percudani and Peracchi, 2003; Milano et al., 2013). Among those, Type I group, the most common structure, is present in aminotransferases, decarboxylases, and enzymes that catalyze α-, β- or γ-eliminations. Type II encode the enzymes involved in β-elimination reactions. Type III is mainly alanine racemase-specific whereas type IV enzymes generally include D-alanine aminotransferase. Type V fold represents the most distinct group among five types and includes mostly glycogen and starch phosphorylases (Percudani and Peracchi, 2003).

Among the PLP-dependent enzymes, type II PLP decarboxylases (henceforth mentioned as ‘PLP_deC’) form an important group of ‘Carboxy-Lyases.’ This group is comprised of L-glutamate decarboxylase (GAD), aromatic L-amino acid decarboxylases (commonly mentioned as AADs/AAADs/AADCs in the published literature), and serine decarboxylase (SDC; group II), prokaryotic forms of ornithine, lysine and arginine decarboxylases (group III), and the eukaryotic forms of ornithine and lysine decarboxylases (group IV; Sandmeier et al., 1994). In this mini-review, we first briefly summarized the available information on their functional roles in plants. Then we highlighted the long prevailed challenges associated with the annotation of PLP_deCs. Unavailability of high quality and comprehensive sequence datasets remained one of the main reasons for their incorrect annotation in the past, therefore we explored their full complement in 52 plant species and analyzed their evolutionary lineage. Taken together, the summary presented here would help us in improving their annotation in large number of plant species; an important factor which has long impeded their rapid functional and biochemical characterization in plants.

## An Overview of the Roles of PLP_deCs in Plant Development

Available expression data indicate that PLP_deCs exhibit developmental, tissue-specific, and inducible transcripts accumulation during plant development (De Luca et al., 1988; Aerts et al., 1992; Pasquali et al., 1992; Facchini et al., 1996; Maldonado-Mendoza et al., 1996; Lopez-Meyer and Nessler, 1997; Liu et al., 2012). In addition, several PLP_deCs have been characterized for their roles in plant development (De Luca et al., 1988; Facchini and De Luca, 1995; Facchini et al., 2000; Torrens-Spence et al., 2014a). For example, GADs, which catalyze the conversion of L-glutamate to γ-aminobutyric acid (GABA); a non-protein amino acid, are involved in a range of cellular processes, including pollen-tube development in *Arabidopsis* and *Picea wilsonii* (Palanivelu et al., 2003; Ling et al., 2013), vascular development in pine (Molina-Rueda et al., 2015), stem elongation (Baum et al., 1996), cytosolic pH regulation, balancing the carbon/nitrogen, defense and protection against biotic and abiotic stresses (Bouche et al., 2004). The main mechanism that contributes to GABA production involves decarboxylation of glutamate via GADs in plants (Akihiro et al., 2008; Takayama and Ezura, 2015). Of these GADs, two GADs, including *SlGAD2* and *SlGAD3* have been identified as the major contributors of GABA conversion in tomato fruits. It has been established that differential activities of these enzymes during fruit development is the main reason of the higher glutamate content in the ripened tomato fruits and their peculiar ‘umami’ taste (Rolin et al., 2000; Carrari and Fernie, 2006; Akihiro et al., 2008; Saito et al., 2008; Osorio et al., 2011). The role of these GADs in determining fruit quality through GABA production may be conserved as increased GAD expression has also been reported during ripening in other fruits such as Chinese berry (*Myrica rubra*; Feng et al., 2012). Further, Ca^2+^/calmodulin (CaM) has been identified as one of the main signaling mediators which are responsible for the conversion of glutamate into GABA. A C-terminal calmodulin-binding domain (CaMBD) in GADs of the majority of plants has been suggested to be both required and responsible for Ca^2+^/CaM-dependent activation of the oligomerized GAD complexes in plants (Zik et al., 2006; Akama and Takaiwa, 2007). However, evidence also suggests that its presence is not universally essential for such activity as several GADs, lacking a typical CaMBD, have been found to function independent of Ca^2+^/CaM in rice and apple (Akama et al., 2001; Fait et al., 2008; Trobacher et al., 2013).

AADs represent the second important category of PLP_deC enzymes which catalyze the decarboxylation of aromatic L-amino acids. These enzymes are mainly involved in the biosynthesis of secondary metabolites in plants (De Luca et al., 1988; Tieman et al., 2006; Lehmann and Pollmann, 2009). The best investigated enzymes in this category are Dopa decarboxylase (DDC), L-tryptophan decarboxylase (TDC), L-tyrosine decarboxylase (TYDC), and histidine decarboxylase (HDC) (Facchini et al., 2000; Torrens-Spence et al., 2014a). Whereas TDC catalyzes decarboxylation of tryptophan to tryptamine and other mono-terpenoid indole alkaloids such as serotonin (5-hydroxytryptamine), TYDC mediates conversion of L-tyrosine to tyramine (Lopez-Meyer and Nessler, 1997; Facchini et al., 2000; Asano et al., 2012). Due to their significance in the production of secondary metabolites, these genes have also been used in the genetic manipulation studies aiming at improving the contents of pharmaceutically important bio-molecules in transgenic plants and/or cell lines, which is summarized in **Table 1**. Similarly, HDC, which catalyzes the conversion of histidine to histamine, has been found to participate in synthesis of the flavor volatiles 2-phenylethanol and 2-phenylacetaldehyde in tomatoes (Picton et al., 1993; Tieman et al., 2006). Their recent characterization in tomato and pepper further identified four HDC ripening-preferential homologs, including *HDC9*, *HDC10*, *HDC11*, and *HDC12*; which may be involved in the similar biochemical conversions to regulate the overall fruit quality (Kumar et al., 2015).

**Table 1:** List of the selected examples where PLP_deCs were undertaken in the genetic manipulation studies.

Target gene	Engineering approach	Phenotype	Plant used	Reference
**Glutamate decarboxylase (GAD)**				
GAD	Overexpression	Higher GABA levels, improved resistance against the root-knot nematode	Tobacco	McLean et al., 2003
GAD	Overexpression	Higher GABA, resistance to tobacco budworm larvae	Tobacco	MacGregor et al., 2003
GAD2	Overexpression	Higher GABA	Rice	Akama and Takaiwa, 2007
Human GAD65	Overexpression	Higher GAD65 content	Tobacco	Avesani et al., 2014
GAD from Petunia	Overexpression	Higher GABA in seeds	*Arabidopsis*	Fait et al., 2011
GAD2	Overexpression	Higher GABA	Rice	Shimajiri et al., 2013
GAD	Suppression	Impede regeneration of transformed explants	Tomato	Chew and Seymour, 2013
GADs	Overexpression, suppression	Altered GABA levels	Tomato	Takayama and Ezura, 2015
**Aromatic L-amino acid decarboxylase (AAD/AADC)**				
TDC from periwinkle	Overexpression	High tryptamine	Tobacco	Poulsen et al., 1994
TDC	Overexpression	Low indole glucosinolates	Canola	Chavadej et al., 1994
TDC from periwinkle	Overexpression	Less tryptamine	Petunia	Thomas et al., 1998
TDC and TyDC	Overexpression	Higher tryptamine and hydroxycinnamic acid amides of tyramine	Tobacco	Guillet et al., 2000; Guillet and De Luca, 2005
TyDC from parsley	Overexpression	Enhanced tyrosol glucaside	Potato	Landtag et al., 2002
TDC1	Overexpression	Higher tryptamine, improved resistance against forest tent caterpillar and tobacco hornworm	Poplar and tobacco	Gill et al., 2003
TyDC2 from poppy	Overexpression	Increase wound-induced tyramine-derived hydroxycinnamic acid amide	Tobacco	Hagel and Facchini, 2005
AADCs	Overexpression	Secondary metabolites	Tomato	Tieman et al., 2006
TDC from *C. acuminata*	Overexpression	Resistance against *Malacosoma disstria*	Poplar	Gill and Ellis, 2006
TDC and TyDC	Overexpression	Altered serotonin and tyramine levels	Rice	Kang et al., 2007; Kang et al., 2009
TyDC	Overexpression	Octoparnine synthesis	Rice	Lee et al., 2010
AtAAS	RNAi	Reduced phenyl acetaldehyde	*Arabidopsis*	Gutensohn et al., 2011
RyAAAT3	RNAi	Lower 2-phenylethanol (PE) content	Rose	Hirata et al., 2012
TyDC	Overexpression	Tyramine overproduction	Rice	Park et al., 2012
TDC	Overexpression	Enhanced serotonin	Rice	Kanjanaphachoat et al., 2012
RcTyDC	Overexpression	Higher tyramine and salidroside content	*Rhodiola crenulata*	Lan et al., 2013
TDC	Overexpression	Enhanced metabolites in cell cultures	*Rauwolfia serpentina*	Mehrotra et al., 2013
TDC	Overexpression	Higher melatonin	Rice	Byeon et al., 2014

PLP_deC members of the third group encode SDC. These enzymes catalyze the conversion of serine to ethanolamine (EA) in plants. EA may act as a precursor of phosphatidylethanolamine (PE) and phosphatidylcholine (PC); the major phospholipids in eukaryotic membranes (Gibellini and Smith, 2010). Similar to the other PLP_deCs, these enzymes also determine the levels of secondary metabolites such as choline in plants (Mudd and Datko, 1989; Rontein et al., 2001).

## Role of PLP_deCs in Stresses

Growing evidences suggest that transcript levels of PLP_deC genes are also influenced by both abiotic (Menke et al., 1999; Lee et al., 2010; Akcay et al., 2012; Liu et al., 2012; Al-Quraan et al., 2013; Hyun et al., 2014; Hu et al., 2015; Kumar et al., 2015) and biotic stresses (Kawalleck et al., 1993; Facchini et al., 1996; Lopez-Meyer and Nessler, 1997; Li et al., 2013; Yogendra et al., 2014). Further, plant hormones such as IAA (Aerts et al., 1992; Goddijn et al., 1992), ABA, salicylic acid, and ethylene (Turano and Fang, 1998; Wang et al., 2000; Kumar et al., 2015) and metal ions Ni^2+^, Mn^2+^, Cu^2+^, Fe^3+^, and Mg^2+^ also modulate their expression (Fujimori and Ohta, 2003; Yang et al., 2013). The elevated GABA levels have been implicated in improving plant survival under abiotic stresses such as salinity and hypoxic conditions in tomato (Yin et al., 2010; Mae et al., 2012). It also improves plant resistance to the northern root-knot nematode in tobacco (McLean et al., 2003), and to fungal pathogens in rice (Forlani et al., 2014). Similarly, TDC mediated enhanced alkaloids production is known to confer resistance in transgenic poplar and tobacco plants against their specific herbivores (Gill et al., 2003). Enhanced amino acid metabolism through transcriptional activation has been proposed to be the underlying molecular mechanisms for such improved tolerance. In this context, transcription factor OsMYB55 has been found to impart its function by regulating *OsGAD3* activity via directly binding to its promoter and activating GABA production under hyperthermia in *OsMYB55*-overexpression transgenic plants (El-Kereamy et al., 2012). Altogether, one of the main roles of PLP_deC enzymes appears to be in stress alleviation via controlling the production of secondary metabolites in plants.

## Challenges Associated with the Identification and Annotation of PLP_deCs in Plants

Plant PLP_deCs share a common evolutionary lineage, however, significant sequence divergence has resulted in an intricate evolutionary relationships between the orthologous enzymes and their functional divergence. Since only a limited number of PLP_deC enzymes have been characterized, till date, elucidation of the complete range of their physiological roles in more plant species remains a monumental task. Their functions have been predicted on the basis of their sequence homology to the already characterized closest PLP_deCs, however, this approach is not always infallible (Thornton et al., 2000). For example, AtSDC was initially characterized as a HDC member (Rontein et al., 2003). A similar survey of the SDC-like protein homologs in GenBank revealed an incorrect annotation of their several homologs as HDC-like/AADs (Torrens-Spence et al., 2013). More recently, we also erroneously annotated tomato AtSDC homolog as SlHDC1 (Kumar et al., 2015). It is noteworthy to mention that upon phylogenetic analysis, SDC-like members were closely placed with HDC proteins in the same major clade and the discrepancies observed in their annotation might have occurred due to the higher sequence similarity between the members of two classes.

Until recently, both SDC and SDC-like enzymes were considered to be functionally conserved. However, new biochemical evidence suggests that these enzymes are functionally diverged in plants as the two SDC-like enzymes in chickpea and *Medicago truncatula* have been found to have unusual aldehyde synthase activity (Torrens-Spence et al., 2014b). Despite acting on the aromatic amino acids, these proteins demonstrated limited homology to the other characterized plant AADs and their preferred substrates were discovered to be the bulky hydrophobic amino acids (Facchini et al., 2000; Lehmann and Pollmann, 2009; Torrens-Spence et al., 2014b). Similar to SDCs, the sequence and the phylogenetic ambiguity between TYDC and TDC members especially that of rice in the previous studies makes annotation of plant AADs challenging (Kumar et al., 2015). In brief, the major challenges associated with the annotation of plant PLP_deCs are, first; presence of multiple gene models predicted in a genome under the same gene model name; second; lack of a high quality genome sequence for a few published draft genomes, third; the diverse substrate preferences of PLP_deC enzymes, fourth; availability of the limited information on their biochemical activities and preferred substrates, and fifth; a high sequence similarity between PLP_deCs especially between HDC and SDC-like proteins and TDCs and TYDCs.

Similarly, as a consequence of the high sequence similarity between TDC and TYDC or HDC and SDC members, elucidation of their preferred substrate specificities is always difficult. The situation gets more complicated by the fact that the activities of TYDCs and TDCs can change by changing a single active site residue; even without altering their substrate selectivity. Substitution of a tyrosine residue to phenylalanine in an active site catalytic loop of plant AADs was found to alter their decarboxylase activity to aldehyde synthase chemistry (Torrens-Spence et al., 2013). Catalytic promiscuity (the ability of a single enzyme to catalyze different chemical reactions) or loose substrate specificity of PLP_deC enzymes augments this situation further, implying that an organism may have more PLP-dependent activities than the actual number of genes, encoding these enzymes. It can also complicate the present scenario regarding their annotation (Percudani and Peracchi, 2003). One of the solutions to this problem lies in computation of newer and better optimized bioinformatics pipelines for the identification of putative active site residues; especially by training them on the available information of the already characterized plant PLP_deCs (De Luca et al., 1988; Facchini and De Luca, 1995; Facchini et al., 2000; Torrens-Spence et al., 2014a). Notably, a similar approach has successfully resulted in the identification of a glycine as the key residue in TDC sequences, whereas a serine occupied the same position in the same conserved motif in the TYDC sequences in *Papaver somniferum* and *Catharanthus roseus* (Torrens-Spence et al., 2014a). Besides the identification of such key residues, four additional residues which did not have any obvious role in governing their indolic or phenolic substrate specificity were also identified. This finding implicated that the activity of plant PLP_deCs is governed by a small number of residues.

## Evolution of PLP_deCs in Plants

**FIGURE 1:**
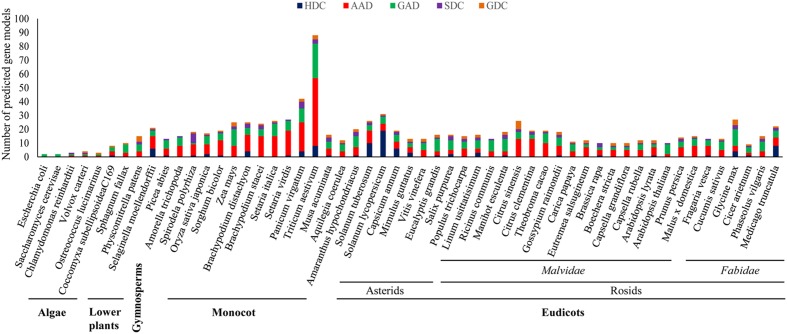
**Identification of PLP_deC genes in 52 plant species, including members of algae, non-vascular lower plants, gymnosperm, and angiosperms, representing all the major clades of the species trees of plants, revealed a clear expansion of these genes from algae to land plants during evolution**. HDC, histidine decarboxylase; AAD, aromatic L-amino acid decarboxylase; GAD, glutamate decarboxylase; SDC, serine decarboxylase; GDC, glycine decarboxylase.

The evolutionary profiling of PLP_deCs using the available sequencing data of plant genomes can further help in the correct identification and functional elucidation of more orthologs in additional plant species. It has been proposed that the number of PLP_deC genes in an organism depends on its adaptation to the specific nutrient sources. With their roles in diverse aspects of plant development and in both abiotic and biotic stress responses, identification of more such genes in additional species and exploration of the coding sequences, especially corresponding to the key residues determining the specific activities of the encoded enzymes or the C-terminal domains such as CaMBD in GADs, among PLP_deC orthologs would help bridge the crucial knowledge gaps existing in current understanding of their precise functions and underlying molecular mechanisms in plants. An investigation of their full complement in 52 plant species, representing the major clades of the species tree of plants, revealed a clear expansion of these genes in land plants over their aquatic ancestors (**Figure 1**). It is conceivable that the sharp expansion in the PLP_deC complements in early land plants was necessitated by the requirement of additional nutrient sources for their successful acclimatization in the new environment. It was observed that green algae such as *Chlamydomonas reinhardtii* and *Volvox carteri* had only three PLP_deCs, without any HDC member. These genes underwent a slight expansion in microalga *Coccomyxa subellipsoideaC169* which resulted in the origin of HDC gene in this member of chlorophyta. Identification of more PLP_deCs in the non-vascular plant such as *Physcomitrella patens* (15) and their further expansion in the earliest vascular plants, such as *Selaginella moellendorffii* (21), suggested that these genes might have contributed to fulfill the additional N requirement. The PLP_deC complement remained similar in gymnosperms (*Picea abies*) and the most basal angiosperm (*Amorella trichopoda*). However, it again showed a noticeable expansion in monocots as 89 such genes were identified in wheat. It is believable that such high number of these genes in wheat may be due to the presence of three genomes in the hexaploid wheat. Besides wheat, a significant expansion of PLP_deC genes also occurred in its tetraploid relative switchgrass (*Panicum virgatum*). However, lack of such expansion in the other tetraploid *Setaria* complicated the evolutionary trends associated with these genes in monocots. Furthermore, over 60% of the total PLP_deCs in wheat and *Setaria* fell in AAD category, suggesting that this class was preferentially retained in this species during evolution.

Altogether, an expansion in the PLP_deC complements evidently favored the evolution of land plants. Generally, GAD and AAD over HDC and SDC members seem to have been preferred for such expansion, except in a few species with more HDC gene copies such as tomato and potato during evolution in plants. Comparatively, more AADs and GADs were identified in monocots than dicots, however, a few dicot species such as *Eucalyptis grandis* in asterids clade and *Ricinus communis*, *Manihot esculenta*, and *Glycine max* in rosids clade also had more GAD members. Closer examination of the C-terminal of the identified GAD proteins revealed that majority of them contained the CaMBD domain. For example, all tomato GADs possessed this domain suggesting that the oligomerized GAD complexes in tomato might be activated only in Ca^2+^/CaM-dependent manner (Zik et al., 2006; Akama and Takaiwa, 2007). However, lack of a typical CaMBD in a few GAD members in both monocots and dicots further suggested that such GAD enzymes might have evolved to function normally even in the absence of a conserved CaMBD domain. Finally, the evolution of HDC genes in land plants is intriguing as this class was found to be the most diverse in term of the strength of their members, which varied from none (in many plant species) to 19 in tomato. Although each class was found to be expanded at least in few monocot and/or dicot species, it remains unclear how these genes would have benefitted these plants and thus warrant further studies.

## Conclusion and Future Prospects

We discussed the evolutionary trends associated with PLP_deC genes in plants. A clear expansion of the members of the different PLP_deC subclasses was found to accompany the evolution of land plants from their aquatic ancesters. Expansion of a certain subclass of PLP_deC genes such as HDC in tomato or other AADs in wheat raised important questions on their relevance and unknown functions in these species. The analysis of their evolutionary profiles presented herein would help to annotate PLP_deC orthologs in more plant species. A combined approach, including the biochemical characterization method, improved computational tools, especially trained on the already characterized PLP_deCs, and information of the 3-D structures of the representatives of each subclass is required to elucidate their precise functions in plants.

## Author Contributions

The author confirms being the sole contributor of this work and approved it for publication.

## Conflict of Interest Statement

The author declares that the research was conducted in the absence of any commercial or financial relationships that could be construed as a potential conflict of interest.
